# Cognitive Distance, Absorptive Capacity and Group Rationality: A Simulation Study

**DOI:** 10.1371/journal.pone.0109359

**Published:** 2014-10-14

**Authors:** Petru Lucian Curşeu, Oleh Krehel, Joep H. M. Evers, Adrian Muntean

**Affiliations:** 1 Department of Organisation Studies, Tilburg University, Tilburg, The Netherlands; 2 Cognitrom, Cluj-Napoca, Romania; 3 Department of Mathematics and Computer Science, Eindhoven University of Technology, Eindhoven, The Netherlands; 4 Department of Mathematics and Computer Science & Institute for Complex Molecular Systems, Eindhoven University of Technology, Eindhoven, The Netherlands; University Toulouse 1 Capitole, France

## Abstract

We report the results of a simulation study in which we explore the joint effect of group absorptive capacity (as the average individual rationality of the group members) and cognitive distance (as the distance between the most rational group member and the rest of the group) on the emergence of collective rationality in groups. We start from empirical results reported in the literature on group rationality as collective group level competence and use data on real-life groups of four and five to validate a mathematical model. We then use this mathematical model to predict group level scores from a variety of possible group configurations (varying both in cognitive distance and average individual rationality). Our results show that both group competence and cognitive distance are necessary conditions for emergent group rationality. Group configurations, in which the groups become more rational than the most rational group member, are groups scoring low on cognitive distance and scoring high on absorptive capacity.

## Introduction

As groups become ubiquitous information processing units in modern organizations, emergent group level cognitive properties received considerable attention in the literature [1]. Empirical evidence supports the claim that collective cognitive structures and collective cognitive competencies emerge from the interplay of individual cognitive structure and competencies during interpersonal interactions [2] [3] [4]. In particular, previous research builds on the group synergy framework [5] [6] and defines group rationality as the rationality gain, as compared to a simple aggregation of group members’ rationalities (e.g., average or best individual rationality), that can be attributed to interpersonal interactions [2] [7]. Groups achieve strong rational synergy if the group as a collective is more rational than the most rational group member, while groups achieve weak rational synergy if the emergent collective rationality is higher than the average rationality of the group members.

Of particular interest for research advancement and practice is strong cognitive/rational synergy, reflecting the extent to which the group as a whole is more rational than the most rational member of the group. Previous research pointed towards the fact that strong synergy is not easily achieved [5] [6] and in rationality terms, groups are often less rational than the most rational member of the group [2] [7]. Finding ways to foster strong group synergy has important implications for managing decision-making groups and has also the potential to extend the research on group cognition, in particular the emergence of collective cognitive competencies.

An important antecedent of emergent group rationality is the initial configuration of group members’ individual rationalities. Cognitive distance as reflected by the detachment of the most rational group member from the rest of the group has a nonlinear association with both weak and strong group synergy [7]. Groups composed of mostly irrational members and one member that scores high on rationality have difficulties in bridging the cognitive distance between the most rational member and the rest of the group and as such are not likely to achieve strong cognitive synergy. Also, empirical research to date shows that average individual rationality within groups has a positive effect on the emergent group level rationality [4] as well as on both weak and strong cognitive synergy [2]. In other words, the more rational the group members are on average, the higher the chance that their group as a whole will achieve both weak and strong cognitive synergy and will make more rational decisions.

Therefore, average individual rationality is a form of group absorptive capacity likely to be beneficial for strong cognitive synergy. The concept of absorptive capacity originates in the general management literature and describes a social system’s (i.e. group, organization) capacity of acquiring, assimilating, generating and transforming knowledge in order to achieve a competitive advantage [8]. Previous literature on organizational absorptive capacity used a variety of proxies to capture the collective capacity of acquiring and using knowledge and in our study we focus on a direct evaluation of individual decision-making competencies. With the two empirical findings at hand, namely that cognitive distance has a nonlinear association with cognitive synergy [7] and average individual rationality (group absorptive capacity) is conducive for cognitive synergy [2] we derive an interesting venue for further research namely to explore whether group’s absorptive capacity influences the shape of the non-linear association between cognitive distance and strong cognitive synergy. The aim of this paper is therefore to investigate in a simulation study, the effects of the interplay between cognitive distance and absorptive capacity on emergent group rationality.

### Cognitive distance and group absorptive capacity

Groups composed of members varying in their task-related knowledge and cognitive competencies often face the issue of bridging these differences in order to reach consensus on a decision or problem solving approach. For disjunctive tasks, in which the task accomplishment depends on the most knowledgeable or competent group member, groups often have to bridge the cognitive gap between the most knowledgeable group member and the rest of the group. Previous empirical research tested models that predict a non-linear association between cognitive distance (defined as the cognitive gap between the most knowledgeable group member and the rest of the group members) and cognitive synergy [7]. For low levels of cognitive distance, the information shared by the most knowledgeable group member (if any at all) is likely to be redundant with the information shared by the rest of the group, therefore there is no potential for cognitive gains or cognitive synergy. As the cognitive distance increases from low to average, at least partially the knowledge held by the individual members of the group becomes non-redundant, while partially they continue to share redundant information. Partially overlapping knowledge repertoires facilitate effective communication and cross-understanding [9]. Results from agent-based simulations also show that convergent opinion adjustment in interacting groups emerges only when original individual opinions share some degree of similarity [10] [11]. Therefore, as the cognitive distance increases from low to average, groups have a higher chance of achieving synergy as the increasing cognitive diversity (the non-redundant information shared by the group members) within the group fosters information elaboration and integration [7] [12]. Nevertheless, if cognitive distance further increases from average to high, it becomes more difficult for the most knowledgeable member to persuade the rest of the group members due to miscommunication and misunderstanding [7]. Moreover, when cognitive distance increases from average to high, it becomes more difficult for the best performing group member to benefit from the added value of the other group members’ specific task-related knowledge. Because of their lack of task-related expertise, the information they share during group debates is either task irrelevant or redundant with the information held by the best performing group member. In motivational terms, according to the Kohler effect [13], when cognitive distance is very large, the best performing individual may also lack motivation to engage with the rest of the group [14] [15].

Building on the above mentioned arguments, Meslec and Curşeu [7] reported two field studies that documented a non-linear association between cognitive distance and both weak and strong cognitive synergy. Their results show an inverted U shape association between cognitive distance and weak cognitive synergy in two types of tasks (judgmental and decision making) as well as an increasing negative association (decelerating) between cognitive distance and strong cognitive synergy in a set of decision tasks. As argued before, the reason for the decelerating relationship between cognitive distance and strong cognitive synergy has a cognitive and a motivational explanation. The motivational explanation resides in the Kohler effect that postulates a decrease in motivation to engage with the group task under marked skills and competencies differences in groups [13]. The cognitive explanation resides in the redundancy of information shared by inexperienced group members during interpersonal interactions as well as their lack of competence in working with the input of the best performing individual in the group.

Meslec and Curşeu [7] used the summed performance across ten decision tasks as an indicator of group and individual rationality. In this particular set of decision tasks, a score of 10 reflect high rationality, while a score of 1 reflects very low rationality. A cognitive distance of 2 points (2 points separate the best performing individual from the average performance of the remaining members) may have different meanings on the 1 to 10 scale. Suppose we have two groups of three members with the following summed individual scores on the decision tasks: Group 1 = (1,1,3) and Group 2 = (6,6,8). In both groups, the cognitive distance computed as the difference between the best score and the average of the rest is 2, yet the dynamics of cognitive emergence is likely to be very different. The first group has insufficient cognitive resources to achieve cognitive synergy in the first place as knowledge is likely to be redundant (the three group members may have solved successfully the same decision task). The second group however, is likely to have a more diverse pool of cognitive resources to draw from and as such a higher cognitive absorptive capacity. A key question thus arises of what happens when the group has enough computational resources to work with and eventually improve the input provided by the best performing individual in the group?

We argue here that groups with high absorptive capacity are better equipped to integrate effectively the knowledge shared by the most knowledgeable group member and as such they are more likely to successfully bridge the cognitive gap that separates the best performing individual in the group from the rest of the group. Such a claim is very difficult to test in real life settings, because it is extremely challenging to find enough groups to cover all possible group configurations in which both cognitive distance and absorptive capacity to vary. We therefore set out to develop a simulation model and test the effects of the interplay of cognitive distance and absorptive capacity on strong and weak cognitive synergy. Computational experiments can capture, starting from existing relations as identified in the empirical studies with real-life groups, relationships between variables and then extrapolate these relations for all possible configurations of the two variables explored. As argued, this extrapolation will never be possible in real life as it is close to impossible to gather data on groups that cover all the spectrum of possible combinations between cognitive distance and absorptive capacity. Starting from real life data reported in previous studies [2] [4] [7] we developed a mathematical model that can effectively predict strong cognitive synergy. We then use the mathematical model to generate results for various group configurations with respect to cognitive distance and absorptive capacity.

## Methods

### Ethics statement

Specific approval from the university ethics review board was not required since this study reports a simulation based experiment and it did not involve additional data collection from human or animal subjects. Data for testing the mathematical model used in the simulation was obtained from previously published studies.

### Data preparation and model testing

With the consent of the authors we used real life data from previously published papers on group rationality [2] [4] [7] as a starting point for our simulation study. In order to make sure we controlled for group size and we have enough raw data to validate the initial model, we selected from all three data bases only groups of four and five members as these group sizes were the most represented in the previously used samples.

### Data pre-processing and the model

The group data is split up by group size, and then groups of different sizes are treated separately in order to control for the co-varying of cognitive distance measure with group size. If we were to investigate groups of all sizes simultaneously, we would have had to contend ourselves with only averaged individual performance and the within group standard deviation, and perhaps the best individual performance in the group to ensure model fit – therefore rely on much less data than in the current approach. Afterwards, the columns for each group are sorted according to the individual performance. We then performed a linear regression of the group score based on the sorted individual scores. For a group of size five, we get five individual coefficients: 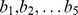 (where 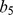 is the coefficient for the best performing individual in the group). Then our estimate of group score g, based on individual scores 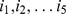 is: 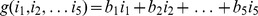. Using this regression model we tested the predictive power of the equation by correlating the “theoretical” scores obtained by using the function inferred from the data with the “true” group scores present in the original data set. This way for each group, each individual group member gets a share of the group score based on his/her position within the group. The equation has very good predictive power, the correlation between the theoretical and true scores being .833 (when a constant is added to the equation the correlation is .839) for groups of five members and .709 (when a constant is added to the equation the correlation is .709) for groups of four members. Given the fact that adding the constant does not substantially increase the predictive power of our model, we decided to use the equation without the constant for the simulation.

Group absorptive capacity and cognitive distance were the independent variables in our simulation study. We used the average individual rationality within groups as a measure of groups’ absorptive capacity and we used a heuristic method of computing cognitive distance. Based on the guidelines reported in Meslec and Curşeu [7] we have computed the cognitive distance as the difference between the highest individual rationality score and the average score for the remaining of the group members. More specifically, for a group of size five, the absorptive capacity is computed as: 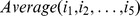, while the cognitive distance is computed as: 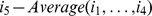, where 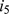 is the highest rationality score in the group and 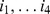 are the rationality scores of the remaining group members.

## Results

**Figure 1:**
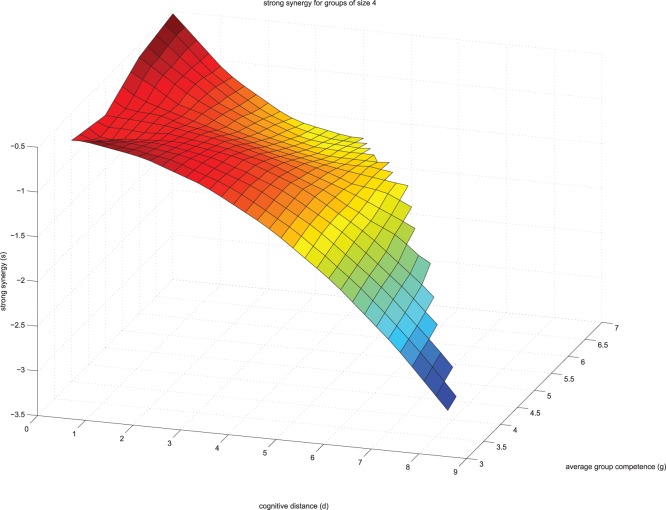
The relationship between cognitive distance and strong cognitive synergy in groups of size 4.

**Figure 2:**
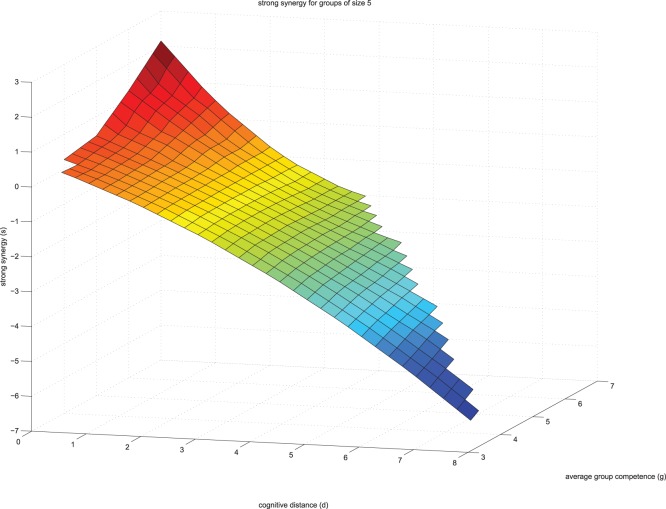
The relationship between cognitive distance and strong cognitive synergy in groups of size 5.

Our simulation was programed and ran in MATLAB version 8.1.0.604 (R2013a). In the supporting information folder (File S1) we present the original data (file labeled S1data.xlsx) as well as the MATLAB syntax used in this study (files labeled: S2analyze.m; S3bucket.m; S4create_by_means.m and S5plot_triples.m). With the fitted coefficients b1, b2, b3 and b4, we make our estimates for all possible group configurations that have their average competence in the range of [3.5,7.5]. We then generated strong synergy scores as a function of average group competence and cognitive distance. For this, we first split our domain of average group performance [3.5,7.5] into smaller sub-regions [3.5, 4.0), [4.0, 4.5), … , [7.0,7.5). Then we generate for each sub-region all possible group combinations that have an average individual performance that falls into this region. We compute for each of these group combinations the expected group rationality, according to the equation coefficients that we derived from real data. We estimated strong synergy for each sub-region as multi-variate function of cognitive distance and group competence. We make a smoothing of this function by fitting it with a quadratic polynomial. We then plot strong synergy as a function of average group competence and cognitive distance. We note that the graphs look qualitatively similar, when we use the three original independent data sets to obtain the fitting. The results of the simulation study are presented in Figures 1 (group size 4) and Figure 2 (group size 5).

As illustrated in both figures, our simulation study replicated the empirical results presented in Meslec and Curşeu [7] for low to average levels of group absorptive capacity. For each fixed average group competence, the strong synergy tends to decrease in a quadratic way, as the cognitive distance increases. For groups scoring high on absorptive capacity, the strong synergy tends to decrease asymptotically as cognitive distance increases. According to our results, only groups scoring low on cognitive distance and high on absorptive capacity achieve strong cognitive synergy, namely the group as a whole is more rational than the most rational group member.

## Discussion

Although common in behavioral ecology, simulation studies starting from real life data [16] [17] [18] [19], are seldom used in disciplines like Social Psychology or Management [20]. Our study provides initial support for using simulation studies to explore group decision making and in particular our results speak to the need of using more computational experiments that extend our understanding of the emergence of collective group level properties, in particular group rationality. We extend the insight of Meslec and Curşeu [7], namely that cognitive distance has a decelerating relationship with strong cognitive synergy in groups and we show that groups’ absorptive capacity tends to change the shape of this relationship. We replicate the results reported in Meslec and Curşeu [7] for low to average levels of group rational competence. At the highest levels of absorptive capacity the association between cognitive distance and strong cognitive synergy has a U shape, with the highest synergy obtained for groups scoring low on cognitive distance and high on absorptive capacity.

According to our simulation results, a necessary condition for achieving strong cognitive synergy is that individual group members score high on rationality. If groups are composed of irrational individuals, they do not have the potential to become more rational than their most rational member. This finding is somehow at odds with studies on animal collectives showing that groups composed of irrational agents can in fact make rational choices [21]. Previous research on collective intelligence reports a rather low correlation between individual level cognitive competences and emergent group level intelligence [22]. Our results show that average individual rationality is positively and strongly associated with the synergetic potential of groups. In other words, according to our simulation results, human groups can be rational only to the extent that their members are rational. In line with the Kohler effect discussed earlier, when cognitive distance is low, the best performing individual member is motivated to engage with the rest of the group and as such strong synergy (as emergent group level rationality) seems to be a function of both individual ability and motivation. An interesting result concerns the emergence of synergy at high levels of absorptive capacity. It seems that as cognitive distance increases from low to average levels, strong cognitive synergy decreases. Nevertheless, and as cognitive distance further increases from average to high, the drop in strong cognitive synergy levels out. To conclude, high average individual rationality is one of the necessary conditions for group rationality to emerge.

Another important result of our simulation study, refers to the association between cognitive distance and group rationality (as strong cognitive synergy). At very low levels of cognitive distance, there is a small positive association between group competence and strong synergy. This pattern of results suggests that low cognitive distance is yet another necessary condition for achieving strong cognitive synergy. To conclude, our simulation study identified two necessary conditions for reaching strong synergy in groups. One of the necessary conditions is high group competence (absorptive capacity) and the other is low cognitive distance. When at least one of these conditions is not fulfilled, groups have little chance of achieving strong synergy that is, becoming more rational than their most rational group member. Yet another configural characteristic conducive for strong synergy is group size and according to our simulation results, at high levels of group absorptive capacity, groups of five have a higher chance of reaching strong cognitive synergy than groups of four. Availability of cognitive resources varies with group size and it is possible that groups of five with high absorptive capacity have more cognitive resources they can use to achieve strong synergy as compared to groups of four. This pattern of results comes at odds with previously reported negative association between group size and strong synergy [2]. One plausible explanation is that group size moderates the positive association between absorptive capacity and strong synergy (the positive association between the absorptive capacity and strong synergy increases with group size). An inverted U shape association between group size and strong synergy is yet another explanation for the apparent inconsistency of these findings. The association between group size and strong synergy is initially positive as cognitive resources increase with group size, nevertheless, as group size further increases, the process losses (coordination problems, social loafing, conflict) associated with large group size may decrease the chance of achieving strong synergy in large groups. As only two group sizes are represented in our simulation, we cannot draw definite conclusions on this plausible inverted U shape relationship between group size and strong synergy. Future computational experiments could explore other configural conditions that influence the emergence of strong cognitive synergy. More complex computational models could also capture patterns of interpersonal interaction in groups [12] [16] [23] [24], or decision rules as they are important factors for achieving strong cognitive synergy.

## Supporting Information

File S1 **In the supplementary material folder labeled File S1, we present the original data file (labeled S1data.xlsx) and the MATLAB syntax is presented in the files: S2analyze.m; S3bucket.m; S4create_by_means.m and S5plot_triples.m.**
(ZIP)
